# Conventional and emerging treatments of astrocytomas and oligodendrogliomas

**DOI:** 10.1007/s11060-022-04216-z

**Published:** 2022-12-25

**Authors:** Tobias Kessler, Jakob Ito, Wolfgang Wick, Antje Wick

**Affiliations:** 1 Clinical Cooperation Unit Neurooncology, German Cancer Consortium (DKTK), German Cancer Research Center (DKFZ), Heidelberg, Germany; 2 Department of Neurology and Neurooncology Program, National Center for Tumor Diseases, Heidelberg University Hospital, Heidelberg, Germany; 3 Neurology Clinic and Neurooncology Program, National Center for Tumor Diseases & DKTK, DKFZ, Im Neuenheimer Feld 400, D-69120 Heidelberg, Germany

**Keywords:** Astrocytoma, Oligodendroglioma, Clinical trials, IDH, Targeted therapy

## Abstract

**Purpose:**

Astrocytomas and oligodendrogliomas are mainly diffuse primary brain tumors harboring a diagnostic and prognostically favorable isocitrate dehydrogenase mutation. They are still incurable besides growing molecular knowledge and therapy options. Circumscribed astrocytomas are also discussed here, although they represent a separate entity despite similarities in the nomenclature.

**Methods:**

We reviewed clinical trials, preclinical approaches as well as guideline recommendations form the major scientific Neuro-Oncology organizations for astrocytomas and oligodendrogliomas according to PRISMA guidelines.

**Results:**

After histopathological diagnosis and eventually a maximal safe resection, patients with good prognostic factors may be followed by magnetic resonance imaging (MRI). If further treatment is necessary, either after diagnosis or at progression, diffuse astrocytomas and oligodendrogliomas are mainly treated with combined radiochemotherapy or maximal safe resection followed by combined radiochemotherapy according to current guidelines based on randomized trials. Circumscribed gliomas like pilocytic astrocytomas, CNS WHO grade 1, or pleomorphic xanthoastrocytomas, CNS WHO grade 2, are often treated with surgery alone. Current approaches for therapy optimization include decision of the best chemotherapy regimen. The *IDH* mutation presents a rational target for small molecule inhibition and immune therapy in diffuse astrocytomas and oligodendrogliomas, while the *BRAF* pathway is frequently mutated and treatable in circumscribed gliomas.

**Conclusion:**

Despite establishment of standard treatment approaches for gliomas that include resection, radio- and chemotherapy, there is a lack of effective treatments for progressive disease. Immune- and targeted therapies are currently investigated.

## Introduction

Relevant advances in the molecular diagnostics of brain tumors have been made in recent years, which led to the inclusion of genetic markers into the current 5th edition of the WHO classification of tumors of the central nervous system [1]. *Isocitrate dehydrogenase (IDH) 1 and 2* mutations, *1p/19q* codeletions and *TP53*/*ATRX* status became mandatory diagnostic and prognostic markers in glioma.

*IDH* mutations define distinct tumors and the diagnosis of astrocytomas and oligodendrogliomas is no longer based on pure histological appearance, but on the presence of an *IDH* mutation (both entities) and on the presence (oligodendroglioma) or absence of 1p/19q codeletion which is often associated with loss of *ATRX* (astrocytoma). This classification allows a better prognostication compared to the earlier histology-based classifications [2].

As a separate group with similarities mainly by the historical terminology, pilocytic astrocytomas are defined by alterations in the MAPK pathway, often characteristic and targetable *BRAF* mutations. In addition to their lower molecular complexity, they are less heterogenous and non-diffuse, allowing for local treatments. Especially surgery, but also radiotherapy, are effective long-term measures to help or even cure patients [3]. Pleomorphic xanthoastrocytomas, CNS WHO grade 2, are often characterized by a *BRAF* V600 mutation [4].

Methylation profiling has recently allowed a more refined diagnosis of brain tumors [5]. *IDH* mutations induce a neomorphic function of the enzyme [6] which leads to hypermethylation and changes in the immune environment [7]. Therefore, *IDH* mutant gliomas show methylation profiles distinct from *IDH* wild-type gliomas. Consequently, the WHO classification defines glioblastoma only in *IDH* wild-type situation, as well as diffuse hemispheric and midline glioma, and groups former secondary glioblastoma into the class of astrocytoma, *IDH* mutant, WHO grade 4 [1].

All the recent advances in molecular understanding have potentially strong implications on current therapy and clinical trials. However, there is still a gap towards routine clinical usage of molecular profiles for clinical decision making. Additionally, most gliomas are not curable today, underlining the need for further treatment development. New targeted- and immune-therapies have entered clinical trials in recent years, offering potential for identifying successful biomarker-drug combinations and treatment stratification based on molecular profiles. This review summarizes the current standard of care for astrocytomas and oligodendrogliomas, highlights recent advances in clinical studies and gives an outlook for future perspectives.

## Methods

### Search strategy

A systematic review following the Preferred Reporting Items for Systematic reviews and Meta-Analyses (PRISMA) 2020 statement was implemented. References were retrieved from the PubMed database using the search terms “glioma”, “astrocytoma”, “oligodendroglioma”, “pilocytic astrocytoma”, “pleomorphic xantoastrocytoma”, “trial”, “clinical”, “surgery”, “radiotherapy”, “chemotherapy” and “immunotherapy” as of August 31st, 2022. Publications between September 1st, 2003, and August 31st, 2022 (span of 20 years) were considered.

Only original research papers with available full text in English were included. Randomized controlled trials (RCT) were included. Case reports, review papers, commentaries, editorials, and meeting abstracts were excluded. Uncontrolled trials were only included in case of high conceptual importance as agreed within the authors. Only studies that included a tumor entity relevant for the scope of this review (astrocytoma, *IDH*-mutant, oligodendroglioma, *IDH*-mutant or astrocytoma that fall in the group of circumscribed gliomas) were included. Furthermore, information on histology and at least *IDH* and 1p/19q status should be described in the publication to allow judgment based on the current WHO classification. For conventional treatments, guidelines of the scientific associations EANO, ASCO, SNO and EURACAN were analyzed for reference and bias reduction [8–10].

Research for the preclinical evidence and emerging treatments section was identified using similar search terms. Clinical and preclinical studies with high translational relevance were included as agreed within the authors.

### Study selection

Initial literature analysis was performed by reviewing titles and abstracts of identified papers and selection based on above mentioned criteria. Full text of relevant articles was extracted. We identified nine completed and three ongoing clinical trials with published study protocols which were included in the analysis (Table 1).

**Table 1:** Completed and ongoing clinical trials for oligodendroglioma and astrocytoma. Trials are listed in alphabetical order. Only trials that fulfilled the criteria in described in search strategy section were included.

Trial	Entity	Treatment	Status	References
CODEL	Oligodendroglioma, WHO grade 3, primary	RT +/- TMZ vs. TMZ	completed	[32]
EORTC 22,033–26,033	Oligodendroglioma/astrocytoma, WHO grade 2, primary	RT vs. TMZ	completed	[22]
EORTC 26,091/TAVAREC	Astrocytoma, WHO grade 2/3, recurrent	TMZ vs. TMZ + BEV	completed	[28]
EORTC 26,951	Oligodendroglioma, WHO grade 3, primary	RT vs. RT + PCV	completed	[31]
EORTC-26,053/CATNON	Astrocytoma, WHO grade 3, primary	RT/(TMZ) vs. RT/(TMZ) + TMZ	completed	[26, 27]
INDIGO	Oligodendroglioma/astrocytoma, WHO grade 2, primary	AG-881 vs. placebo	ongoing	[41]
NOA-04	Oligodendroglioma/astrocytoma, WHO grade 3, primary	PCV or TMZ vs. RT	completed	[25]
NOA-16	Astrocytoma, WHO grade 3/4, primary	RT + TMZ vs. RT + TMZ + IDH vaccination	completed	[37]
NOA-18 / IMPROVE-CODEL	Oligodendroglioma, WHO grade 2/3, primary	TMZ/CCNU ◊ RT + PCV vs. RT + PCV ◊ BPC	ongoing	[34]
NOA-21 / AMPLIFY-NEOVAC	Oligodendroglioma/astrocytoma WHO grade 2/3/4, recurrent	IDH vaccination vs. ICI vs. IDH vaccination + ICI	ongoing	[33]
RTOG 9402	Oligodendroglioma, WHO grade 3, primary	RT vs. RT + PCV	completed	[30]
RTOG 9802	Oligodendroglioma/astrocytoma WHO grade 2, primary	RT vs. RT + PCV	completed	[21]

*RT* Radiotherapy,*TMZ* Temozolomide,*PCV* Procarbacine,*CCNU* Vincristine,*BEV*Bevacizumab,*ICI* Immune checkpoint inhibitor

## Results

### Molecular features of astrocytoma and oligodendroglioma

According to the current WHO classification astrocytomas and oligodendrogliomas are defined as *IDH1/2* mutant tumors, with the exemption of circumscribed gliomas that include entities like pilocytic astrocytoma and pleomorphic xanthoastrocytoma as well as pediatric type gliomas [1]. The most common *IDH* mutation is the *IDH1*(R132H) mutation. Oligodendrogliomas are defined by 1p/19q codeletion, while astrocytomas are 1p/19q non-codeleted tumors with ATRX loss. The term oligoastrocytoma is not in use anymore since morphologically mixed tumors can regularly be assigned to either astrocytoma or oligodendroglioma based on the above-mentioned molecular markers [11].

**Fig. 1:**
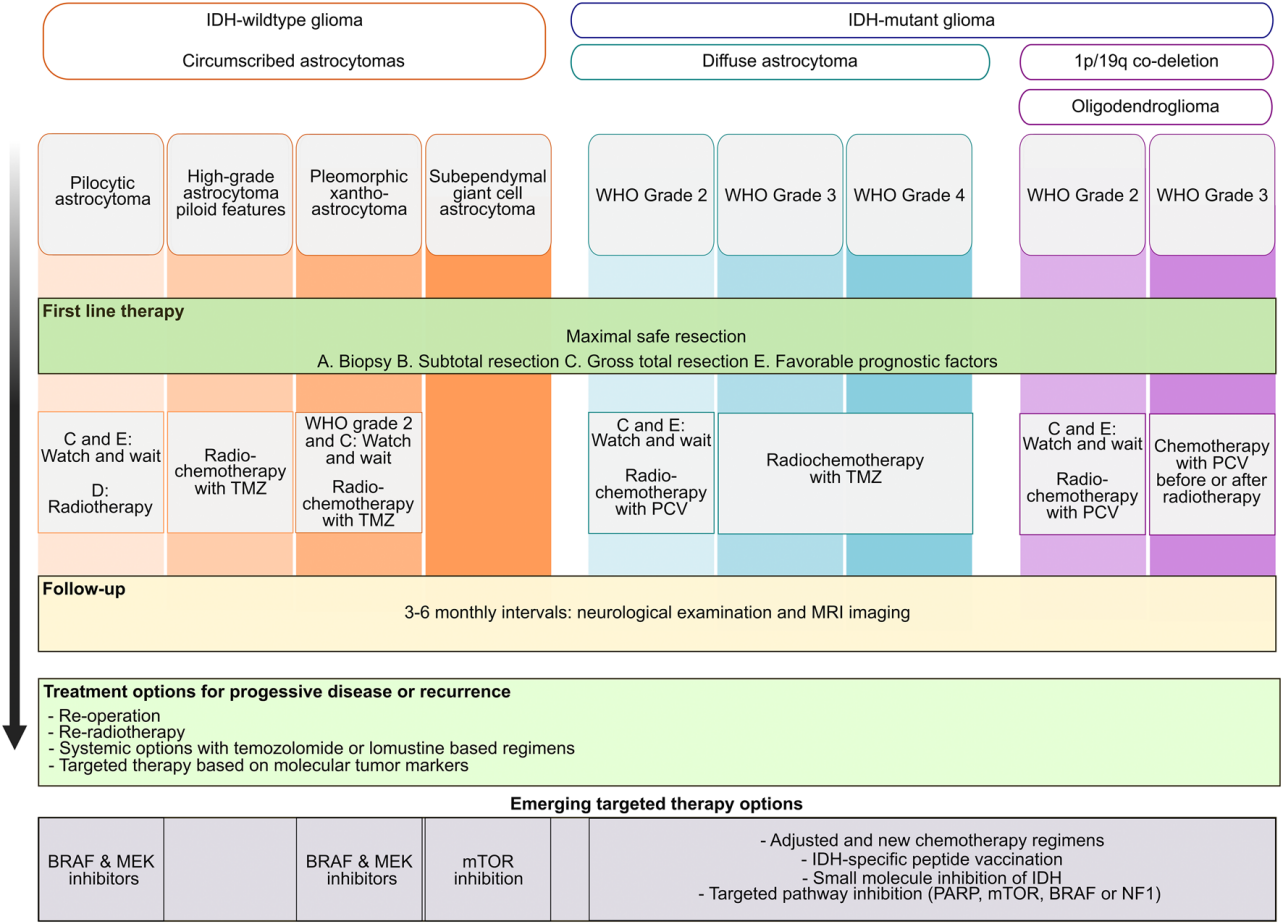
Treatment of astrocytomas and oligodendrogliomas. In this figure, diagnostic groups and classifications, differential first line therapy and potential targeted and emerging treatments are summarized. *TMZ* temozolomide, *PCV *Procarbacine, *CCNU *vincristine

In the next sections, treatment options in the three groups of circumscribed and diffuse astrocytomas as well as oligodendrogliomas are described (Fig. 1). Pediatric type low grade gliomas are not covered in this review. Furthermore, according to the recent WHO classification, diffuse gliomas without *IDH* mutation are not covered as they predominantly harbor molecular features of glioblastoma.

### Current standard of treatment for astrocytomas and oligodendrogliomas

#### Circumscribed astrocytomas

The recent WHO classification subclassifies circumscribed astrocytomas in pilocytic astrocytoma, high-grade astrocytoma with piloid features (HGAP), pleomorphic xanthoastrocytoma and subependymal giant cell astrocytoma. Recently, guidelines for this new group were published by EANO/EURACAN/SNO [10]. Pilocytic astrocytomas show a low rate of malignant progression. Main treatment is surgical resection. Radiotherapy of the tumor region is advised if there are no surgical options [12] and if there is radiographic evidence of progression. Pilocytic astrocytomas harbor alterations in the MAPK signaling pathway [13] which can be regarded as a therapeutic avenue for patients where neither operation nor radiotherapy is a viable option. Pending on the exact molecular alteration, there are pharmacologic approaches with selective BRAF and MEK inhibitors [14]. In the extremely rare instance of HGAP, patients undergo surgical intervention followed by radiochemotherapy [15, 16].

Pleomorphic xanthoastrocytomas, CNS WHO grade 2, are resected microsurgically. Radiotherapy may be indicated for patients with partial resection, tumor progression, or inoperability. Pharmacologic inhibition of the BRAF pathway when a *BRAF* V600 mutation is detected and parallel inhibition of MEK to prevent rapid development of resistance appears to be helpful for some patients [17] and is currently under controlled clinical evaluation. Pleomorphic xanthoastrocytoma with CNS WHO grade 3 are treated similarly to astrocytoma, *IDH* mutant with resection followed by sequential radio-chemotherapy with temozolomide. In tumors with CNS WHO grade 2, radiochemotherapy can be delayed to the moment of progressive disease as surgery is potentially curative. The frequent BRAF V600E mutation offers the possibility for combined BRAF/MEK inhibition [14]. Subependymal giant cell astrocytomas typically arise in patients with *TSC1/2* mutation. These tumors show responses to mechanistic target of rapamycin (mTOR) inhibition with everolimus in regards of tumor growth and seizure control [18], and thus everolimus has been approved by FDA and EMA. Following full dose treatment, a reduced maintenance dose of everolimus appears reasonable to keep tumor stability [19].

#### Diffuse astrocytoma, IDH-mutant

The mainstay of treatment for CNS WHO grade 2 diffuse astrocytoma is surgical resection [20], however this is not curative. Younger patients below age 40–45 that are asymptomatic besides having controllable seizures, can be followed up after full surgical resection. Patients above this age or with incomplete resection should be postoperatively receive radio-chemotherapy. Whereby the criterion of “age over 40” is a soft criterion, which will not necessarily mean to begin with the therapy in any case. The RTOG 9802 study showed a survival benefit of radiotherapy combined with procarbazine, lomustine, vincristine (PCV)-chemotherapy compared to radiotherapy alone [21]. However, treatment of patients with *IDH*-mutant astrocytoma with temozolomide alone is probably inferior to radiotherapy based on data from the EORTC 22,033–26,033 trial [22]. Radiotherapy with PCV is now considered standard of care. Although randomized controlled trials are missing temozolomide is often used based on its good safety profile and convenient application. Radiotherapy can be administered in dosages between 45 and 54 Gy based on two clinical trials that did not show a difference between these and higher dose regimens [23, 24].

For patients with CNS WHO grade 3 diffuse astrocytoma maximal safe resection followed by radiochemotherapy is considered standard of care. In the NOA-04 trial, there was no relevant difference between PCV or temozolomide compared to radiotherapy alone [25]. The CATNON (EORTC-26,053) trial showed that radiotherapy followed by up to 12 cycles of temozolomide prolongs overall survival in *IDH*-mutant tumors [26]. Concomitant temozolomide given together with radiotherapy has no relevant further impact [27].

Diffuse astrocytoma, IDH-mutant, CNS WHO grade 4 represents a new entity, without specific studies available. It is expected that those tumors were covered in multiple clinicals trials for glioblastoma as well as anaplastic astrocytoma. Accordingly, treatment consists of radiotherapy with temozolomide after maximal safe resection.

At time of progression, similar treatments as in the primary state of disease including re-operation, re-radiotherapy, and systemic regimens with temozolomide or lomustine are an option, however newer studies are missing that take also current standard first line therapy into account. Bevacizumab does not prolong progression-free or overall survival in patients with *IDH*-mutant astrocytoma in the TAVAREC trial [28] but could be a potential treatment in cases of symptomatic radionecrosis [29].

#### Oligodendroglioma, IDH-mutant, 1p/19q codeleted

Oligodendrogliomas are classified into WHO Grade 2 and 3. Operation is the first and main treatment for WHO grade 2 oligodendrogliomas. Considerations regarding different postoperative approaches, namely watch and wait and adjuvant therapy in patients with tumors are managed similarly to astrocytoma, with the exemption that asymptomatic tumors with incomplete resection can be followed up initially. For adjuvant treatment, patients are treated with radiotherapy and 4–6 subsequent cycles of PCV chemotherapy based on the RTOG 9802 trial [21].

For patients with CNS WHO grade 3 oligodendrogliomas two randomized controlled trials showed a survival benefit of administering PCV chemotherapy before or after radiotherapy following surgical resection [30, 31]. Chemotherapy with temozolomide alone seems to be inferior to radiotherapy or the combination of radiotherapy with temozolomide based on results of the original CODEL trial [32]. Whether temozolomide instead of PCV upfront together with radiotherapy is an option for patients with WHO grade 2 or 3 tumors is currently evaluated in the revised CODEL trial and discussed below. Treatment options at the time of progression include re-operation, re-radio- and chemotherapy and clinical trials using the *IDH* R132H vaccine [33].

### Emerging treatments

#### Improving chemotherapeutic options

Ongoing clinical trials aim at improving the prognosis of patients with astrocytoma and oligodendroglioma and to reduce the burden of the therapy in patients with favorable prognosis. As oligodendrogliomas are rather chemosensitive, current trials focus on improving the chemotherapy regimen especially for 1p/19q codeleted tumors.

The IMPROVE-CODEL/NOA-18 trial aspires to prove the superiority of lomustine and temozolomide (CETEG) plus RT-PCV at progression over RT-PCV as determined at the level of overall survival without sustained functional deterioration for patients with oligodendroglioma [34]. The combination of lomustine and temozolomide has been proven to be beneficial over temozolomide in patients with *MGMT* promotor methylated glioblastoma in the NOA-09/CETEG trial [35] and is currently evaluated in patients with 1p/19q codeletion. The target is to explore the potential of delaying radiotherapy resulting in beneficial effects on quality of life in a population with rather good prognosis. The redefined CODEL trial [32] is now targeting to investigate whether PCV can be substituted by temozolomide which has the advantage of easier administration and a favorable side effect profile.

#### Targeting IDH

One common genetic hallmark of diffuse astrocytomas and oligodendrogliomas is the *IDH*-mutation, which is presented on the major histocompatibility complex (MHC) class II [36], making mutant *IDH* a potential target for immunotherapy and suitable for vaccination approaches. The single arm NOA-16 trial evaluated an *IDH*-specific peptide vaccination for astrocytoma with *IDH1* R132H mutation in combination with radiotherapy and temozolomide for newly diagnosed patients [37]. The study met the safety endpoint and immune responses were observed for most patients. Currently recruiting is the AMPLYFY-NEOVAC trial, assessing the *IDH*-specific vaccination alone or in combination with the checkpoint inhibitor avelumab against avelumab alone in a randomized comparative trial design [33].

Other approaches use small molecule inhibition of *IDH*. The *IDH* inhibitors enasidenib and ivosidenib were approved for treatment of *IDH1/2* mutant acute myeloid leukemia [38, 39]. Preclinical studies showed delayed tumor growth through small molecule *IDH1* inhibition in glioma cells [40]. In a phase I trial, the brain penetrant *IDH1/2* inhibitor vorasidenib showed preliminary activity in progressive *IDH* mutant glioma [41]. The ongoing INDIGO trial evaluates vorasidenib against placebo in primary astrocytoma and oligodendroglioma patients with small lesions that would otherwise undergo watch and wait strategy [42].

#### Further molecular target-based treatment options

Molecular profile based individual treatment decisions after discussion in molecular tumor boards have been established in oncology [43, 44], and are particularly used in neuro oncology for patient cases of rare entities or progressive situations where no treatment options are available [45]. The drugs used are often repurposed from other indications, administered according to similar genetic vulnerabilities, and ranked in an order based on evidence levels [46]. For astrocytomas and oligodendrogliomas based on the specific *IDH* mutation, PARP inhibitors are considered an option. *IDH* mutations can compromise the base excision repair system which could render tumors sensitive to PARP inhibitors [47] if the tumor harbors a *TP53* wild-type gene [48]. However, a phase II study did not show a benefit of the PARP inhibitor veliparib in patients with non *IDH* mutated glioblastoma given in addition to temozolomide [49], but trial results of ongoing studies for *IDH* mutant glioma are pending [50]. Further potential molecular based treatment options that have been investigated in *IDH* mutant glioma include the PI3K/mTOR signaling pathway that could be targeted with mTOR inhibition [51]. In low grade glioma with activation of the MEK/ERK pathway, aberrations in BRAF or NF1 MEK inhibition with trametinib or selumetinib revealed promising activity in pediatric patients [52, 53].

### Preclinical evidence and further perspective

There is an increasing interest in immunotherapies for astrocytomas and oligodendrogliomas. However, preclinical studies specifically designed for *IDH* mutant gliomas are rare, based on the difficulty of culturing *IDH* mutant glioma cells in preclinical models. Preclinical studies evaluated *IDH* inhibition together with immune checkpoint inhibition (ICI) to enhance efficacy of adoptive T-cell transfer of mice vaccinated with a IDH1-R132H peptide [7] or peptides derived from glioma-associated antigens [54]. Inhibition of IDH + ICI prolonged survival of mice compared to *IDH* inhibition or ICI alone which may translate into a promising rationale for the combination arm in the APLIFY-NEOVAC trial. Furthermore, inhibition of 2-hydroxyglutarate mediated immunosuppression with an AhR inhibitor in combination with ICI increased overall survival in mice with *IDH* mutant glioma [55].

Further prospective therapies could be derived from developments in *IDH* wildtype gliomas. Cell therapies have gained high interest in recent years. Especially chimeric antigen receptor (CAR) T cells are being assessed in clinical trials for glioma patients. GD2-CAR T cells showed promising results in three patients with H3K27M-mutated diffuse midline gliomas [56]. However, there is currently limited evidence for *IDH* mutant tumors being significantly affected by GD2-CAR T therapy.

## Conclusion

Advances in molecular diagnostics have increased the biological knowledge of astrocytomas and oligodendrogliomas in recent years. A substantial number of large clinical trials have been completed and established the role of chemotherapy in addition to resection and radiotherapy in a multimodal treatment approach for gliomas. Several questions for further studies remain.

Of high importance is the unknown optimal chemotherapy regimen for diffuse gliomas. Temozolomide is an easy to administer option with a low side effect profile, but the equivalence to PCV has not been shown for oligodendrogliomas and grade 2 astrocytomas. WHO grade 4 astrocytomas present a new entity and it is currently unclear whether these tumors should be treated with concomitant temozolomide [57] or adjuvant temozolomide only [27]. Besides this, the role of upfront combinatorial chemotherapy without early radiotherapy for chemosensitive oligodendroglioma is currently being answered in the IMPROVE-CODEL trial [34]. However, these combinatorial treatments are not curative for most patients and progression is likely at some point in time. At tumor progression, established treatment options are limited to re-operation, re-radiotherapy, and chemotherapy with a lack of clearly effective treatment strategies at this stage.

New targeted advances with small molecules and immunotherapies currently focus on addressing the *IDH* mutation, one of the genetic hallmarks of astrocytoma and oligodendroglioma. Immunotherapeutic treatments are new promising approaches, but they have yet to overcome critical issues including the immunosuppressive tumoral microenvironment in the brain [58]. Finally, the overall effect of drugs used for molecular based targeted therapies as monotherapy, even when selected for high confidence targets, has often been low. Future clinical trials certainly have to evaluate rational combinatory therapies while balancing potential side effects.

## References

[1] Louis DN, Perry A, Wesseling P (2021). The 2021 WHO classification of tumors of the central nervous system: a summary. Neuro Oncol.

[2] Cancer Genome Atlas Research Network, Brat DJ, Verhaak RG, Aldape KD et al (2015) Comprehensive, integrative genomic analysis of diffuse lower-grade gliomas. N Engl J Med 372:2481–2498. 10.1056/NEJMoa1402121 PMC4530011 26061751

[3] Salles D, Santino SF, Ribeiro DA (2022). The involvement of the MAPK pathway in pilocytic astrocytomas. Pathol Res Pract.

[4] Dias-Santagata D, Lam Q, Vernovsky K (2011). BRAF V600E mutations are common in pleomorphic xanthoastrocytoma: diagnostic and therapeutic implications. PLoS ONE.

[5] Capper D, Jones DTW, Sill M (2018). DNA methylation-based classification of central nervous system tumours. Nature.

[6] Pirozzi CJ, Yan H (2021). The implications of IDH mutations for cancer development and therapy. Nat Rev Clin Oncol.

[7] Bunse L, Pusch S, Bunse T (2018). Suppression of antitumor T cell immunity by the oncometabolite (R)-2-hydroxyglutarate. Nat Med.

[8] Weller M, van den Bent M, Preusser M (2021). EANO guidelines on the diagnosis and treatment of diffuse gliomas of adulthood. Nat Rev Clin Oncol.

[9] Mohile NA, Messersmith H, Gatson NT (2022). Therapy for diffuse astrocytic and oligodendroglial tumors in adults: ASCO-SNO Guideline. J Clin Oncol.

[10] Ruda R, Capper D, Waldman AD (2022). Eano-Euracan-Sno Guidelines on circumscribed astrocytic GLIOMAS, Glioneuronal and Neuronaltumors. Neuro Oncol.

[11] Sahm F, Reuss D, Koelsche C (2014). Farewell to oligoastrocytoma: in situ molecular genetics favor classification as either oligodendroglioma or astrocytoma. Acta Neuropathol.

[12] Brown PD, Buckner JC, O’Fallon JR (2004). Adult patients with supratentorial pilocytic astrocytomas: a prospective multicenter clinical trial. Int J Radiat Oncol Biol Phys.

[13] Jones DT, Hutter B, Jager N (2013). Recurrent somatic alterations of FGFR1 and NTRK2 in pilocytic astrocytoma. Nat Genet.

[14] Wen PY, Stein A, van den Bent M (2022). Dabrafenib plus trametinib in patients with BRAF(V600E)-mutant low-grade and high-grade glioma (ROAR): a multicentre, open-label, single-arm, phase 2, basket trial. Lancet Oncol.

[15] Reinhardt A, Stichel D, Schrimpf D (2018). Anaplastic astrocytoma with piloid features, a novel molecular class of IDH wildtype glioma with recurrent MAPK pathway, CDKN2A/B and ATRX alterations. Acta Neuropathol.

[16] Bender K, Perez E, Chirica M (2021). High-grade astrocytoma with piloid features (HGAP): the Charite experience with a new central nervous system tumor entity. J Neurooncol.

[17] Berzero G, Bellu L, Baldini C (2021). Sustained tumor control with MAPK inhibition in BRAF V600-Mutant adult glial and glioneuronal tumors. Neurology.

[18] Krueger DA, Care MM, Holland K (2010). Everolimus for subependymal giant-cell astrocytomas in tuberous sclerosis. N Engl J Med.

[19] Bobeff K, Krajewska K, Baranska D (2021). Maintenance therapy with everolimus for subependymal giant cell astrocytoma in patients with tuberous sclerosis - final results from the EMINENTS Study. Front Neurol.

[20] Jakola AS, Skjulsvik AJ, Myrmel KS (2017). Surgical resection versus watchful waiting in low-grade gliomas. Ann Oncol.

[21] Buckner JC, Shaw EG, Pugh SL (2016). Radiation plus Procarbazine, CCNU, and Vincristine in Low-Grade Glioma. N Engl J Med.

[22] Baumert BG, Hegi ME, van den Bent MJ (2016). Temozolomide chemotherapy versus radiotherapy in high-risk low-grade glioma (EORTC 22033–26033): a randomised, open-label, phase 3 intergroup study. Lancet Oncol.

[23] Breen WG, Anderson SK, Carrero XW (2020). Final report from intergroup NCCTG 86-72-51 (Alliance): a phase III randomized clinical trial of high-dose versus low-dose radiation for adult low-grade glioma. Neuro Oncol.

[24] Karim AB, Maat B, Hatlevoll R (1996). A randomized trial on dose-response in radiation therapy of low-grade cerebral glioma: European Organization for Research and Treatment of Cancer (EORTC) Study 22844. Int J Radiat Oncol Biol Phys.

[25] Wick W, Roth P, Hartmann C (2016). Long-term analysis of the NOA-04 randomized phase III trial of sequential radiochemotherapy of anaplastic glioma with PCV or temozolomide. Neuro Oncol.

[26] van den Bent MJ, Baumert B, Erridge SC (2017). Interim results from the CATNON trial (EORTC study 26053 – 22054) of treatment with concurrent and adjuvant temozolomide for 1p/19q non-co-deleted anaplastic glioma: a phase 3, randomised, open-label intergroup study. Lancet.

[27] van den Bent MJ, Tesileanu CMS, Wick W (2021). Adjuvant and concurrent temozolomide for 1p/19q non-co-deleted anaplastic glioma (CATNON; EORTC study 26053 – 22054): second interim analysis of a randomised, open-label, phase 3 study. Lancet Oncol.

[28] van den Bent MJ, Klein M, Smits M (2018). Bevacizumab and temozolomide in patients with first recurrence of WHO grade II and III glioma, without 1p/19q co-deletion (TAVAREC): a randomised controlled phase 2 EORTC trial. Lancet Oncol.

[29] Fleischmann DF, Jenn J, Corradini S (2019). Bevacizumab reduces toxicity of reirradiation in recurrent high-grade glioma. Radiother Oncol.

[30] Cairncross G, Wang M, Shaw E (2013). Phase III trial of chemoradiotherapy for anaplastic oligodendroglioma: long-term results of RTOG 9402. J Clin Oncol.

[31] van den Bent MJ, Brandes AA, Taphoorn MJ (2013). Adjuvant procarbazine, lomustine, and vincristine chemotherapy in newly diagnosed anaplastic oligodendroglioma: long-term follow-up of EORTC brain tumor group study 26951. J Clin Oncol.

[32] Jaeckle KA, Ballman KV, van den Bent M (2021). CODEL: phase III study of RT, RT + TMZ, or TMZ for newly diagnosed 1p/19q codeleted oligodendroglioma. Analysis from the initial study design. Neuro Oncol.

[33] Bunse L, Rupp AK, Poschke I (2022). AMPLIFY-NEOVAC: a randomized, 3-arm multicenter phase I trial to assess safety, tolerability and immunogenicity of IDH1-vac combined with an immune checkpoint inhibitor targeting programmed death-ligand 1 in isocitrate dehydrogenase 1 mutant gliomas. Neurol Res Pract.

[34] Wick A, Sander A, Koch M (2022). Improvement of functional outcome for patients with newly diagnosed grade 2 or 3 gliomas with co-deletion of 1p/19q - IMPROVE CODEL: the NOA-18 trial. BMC Cancer.

[35] Herrlinger U, Tzaridis T, Mack F (2019). Lomustine-temozolomide combination therapy versus standard temozolomide therapy in patients with newly diagnosed glioblastoma with methylated MGMT promoter (CeTeG/NOA-09): a randomised, open-label, phase 3 trial. Lancet.

[36] Schumacher T, Bunse L, Pusch S (2014). A vaccine targeting mutant IDH1 induces antitumour immunity. Nature.

[37] Platten M, Bunse L, Wick A (2021). A vaccine targeting mutant IDH1 in newly diagnosed glioma. Nature.

[38] Stein EM, DiNardo CD, Pollyea DA (2017). Enasidenib in mutant IDH2 relapsed or refractory acute myeloid leukemia. Blood.

[39] DiNardo CD, Stein EM, de Botton S (2018). Durable remissions with Ivosidenib in IDH1-Mutated relapsed or refractory AML. N Engl J Med.

[40] Rohle D, Popovici-Muller J, Palaskas N (2013). An inhibitor of mutant IDH1 delays growth and promotes differentiation of glioma cells. Science.

[41] Mellinghoff IK, Penas-Prado M, Peters KB (2021). Vorasidenib, a dual inhibitor of mutant IDH1/2, in recurrent or progressive glioma; results of a first-in-human phase I Trial. Clin Cancer Res.

[42] Mellinghoff IK, Bent MJVD, Clarke JL (2020). INDIGO a global randomized double-blind phase III study of vorasidenib VOR; AG-881 vs placebo in patients pts with residual or recurrent grade II glioma with an isocitrate dehydrogenase 1/2 IDH1/2 mutation. J Clin Encol.

[43] van der Velden DL, Hoes LR, van der Wijngaart H (2019). The drug rediscovery protocol facilitates the expanded use of existing anticancer drugs. Nature.

[44] Rodon J, Soria JC, Berger R (2019). Genomic and transcriptomic profiling expands precision cancer medicine: the WINTHER trial. Nat Med.

[45] Kessler T, Berberich A, Casalini B (2020). Molecular profiling-based decision for targeted therapies in IDH wild-type glioblastoma. Neurooncol Adv.

[46] Wick W, Kessler T (2018). Drug Repositioning meets Precision in Glioblastoma. Clin Cancer Res.

[47] Sulkowski PL, Corso CD, Robinson ND (2017). 2-Hydroxyglutarate produced by neomorphic IDH mutations suppresses homologous recombination and induces PARP inhibitor sensitivity. Sci Transl Med.

[48] Sizemore ST, Mohammad R, Sizemore GM (2018). Synthetic lethality of PARP inhibition and Ionizing Radiation is p53-dependent. Mol Cancer Res.

[49] Sim HW, McDonald KL, Lwin Z (2021). A randomized phase II trial of veliparib, radiotherapy, and temozolomide in patients with unmethylated MGMT glioblastoma: the VERTU study. Neuro Oncol.

[50] Sim HW, Galanis E, Khasraw M (2022). PARP inhibitors in glioma: a review of Therapeutic Opportunities. Cancers.

[51] Wahl M, Chang SM, Phillips JJ (2017). Probing the phosphatidylinositol 3-kinase/mammalian target of rapamycin pathway in gliomas: a phase 2 study of everolimus for recurrent adult low-grade gliomas. Cancer.

[52] Fangusaro J, Onar-Thomas A, Young Poussaint T (2019). Selumetinib in paediatric patients with BRAF-aberrant or neurofibromatosis type 1-associated recurrent, refractory, or progressive low-grade glioma: a multicentre, phase 2 trial. Lancet Oncol.

[53] Perreault S, Larouche V, Tabori U (2019). A phase 2 study of trametinib for patients with pediatric glioma or plexiform neurofibroma with refractory tumor and activation of the MAPK/ERK pathway: TRAM-01. BMC Cancer.

[54] Kadiyala P, Carney SV, Gauss JC (2021). Inhibition of 2-hydroxyglutarate elicits metabolic reprogramming and mutant IDH1 glioma immunity in mice. J Clin InvestDoi.

[55] Friedrich M, Sankowski R, Bunse L (2021). Tryptophan metabolism drives dynamic immunosuppressive myeloid states in IDH-mutant gliomas. Nat Cancer.

[56] Majzner RG, Ramakrishna S, Yeom KW (2022). GD2-CAR T cell therapy for H3K27M-mutated diffuse midline gliomas. Nature.

[57] Stupp R, Mason WP, van den Bent MJ (2005). Radiotherapy plus concomitant and adjuvant temozolomide for glioblastoma. N Engl J Med.

[58] Grabowski MM, Sankey EW, Ryan KJ (2021). Immune suppression in gliomas. J Neurooncol.

